# From audit to applause: an antimicrobial stewardship hospital-based prescriber recognition initiative

**DOI:** 10.1017/ash.2026.10900

**Published:** 2026-10-05

**Authors:** Michael Trisler, Robert Volosky, Louise-Marie Oleksiuk

**Affiliations:** 1 UPMC Shadyside, USA; 2 UPMC, Pittsburgh, USA

## Abstract

We implemented a positive reinforcement framework recognizing prescribers for demonstrating independent antimicrobial stewardship practices. In 2025, 222 prescriber actions were recognized, most commonly for avoidance/discontinuation of unnecessary antibiotics for asymptomatic bacteriuria. Most surveyed prescribers felt that the antimicrobial stewardship recognition fostered a more positive relationship with the antimicrobial stewardship program.

## Introduction

Antimicrobial stewardship programs (ASPs) are essential for optimizing antimicrobial use, improving patient outcomes, and mitigating antimicrobial resistance within hospital settings.^1,2^ Core strategies traditionally emphasize prospective audit and feedback, preauthorization, and education, often focusing on identifying and correcting suboptimal prescribing practices.^1^ While these approaches are effective, they are frequently perceived by prescribers as corrective or restrictive, which may contribute to disengagement or resistance despite shared goals.^3,4^

Positive reinforcement and recognition are well-established strategies for influencing professional behavior in healthcare; however, with the exception of peer comparison “report cards,” they are less commonly incorporated into antimicrobial stewardship interventions.^2,5–7^ Recognizing exemplary prescribing practices may bolster prescriber engagement, reinforce desired behaviors, and promote a culture of shared accountability for optimal antimicrobial use.^7–8^

For these reasons, we developed a hospital-based initiative that documents independent stewardship practices and recognizes prescribers on a quarterly basis. This approach aimed to complement traditional strategies by highlighting and reinforcing high-value prescribing decisions already occurring in routine clinical care. We also conducted a prescriber survey to assess clinician perceptions of this recognition initiative. Here, we describe the design, implementation, and impact of this recognition framework as an innovative strategy to support antimicrobial stewardship through positive reinforcement.

## Methods

**Figure 1.**
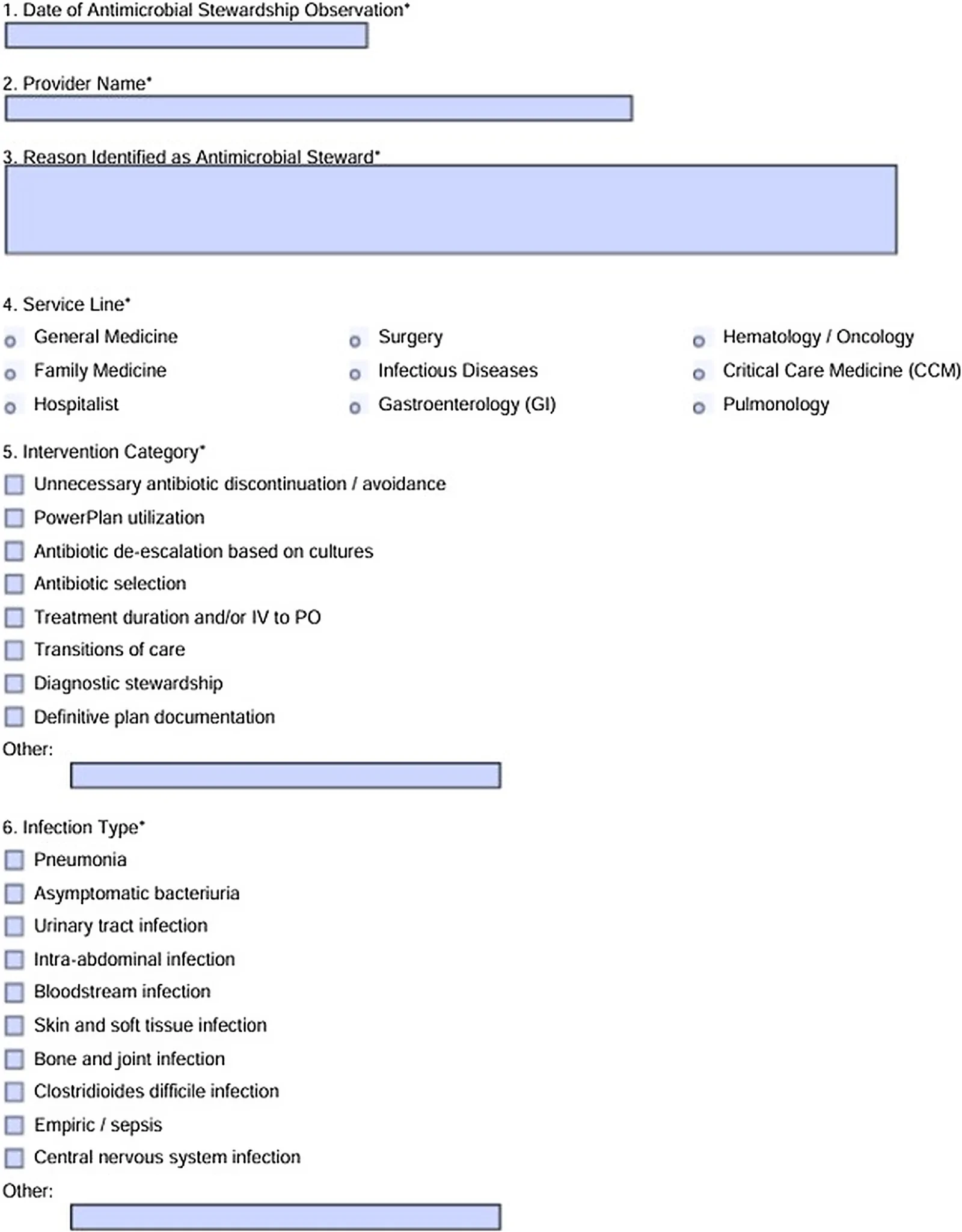
Template for Microsoft Form used to document independent prescriber actions. This image was generated using Microsoft Copilot (Microsoft Corporation, Redmond, WA, USA) and reviewed, edited, and approved by the authors.

This initiative was conducted at an acute care 520-bed hospital with an established ASP from January 1 through December 31, 2025. Our ASP is co-led by an infectious diseases physician and two infectious diseases pharmacists and utilizes both prior authorization (eg, carbapenems, novel gram-negative agents) and prospective audit and feedback (eg, microbiology results, 48-hour timeouts for broad-spectrum antibiotics, and indication-based alerts) to improve antimicrobial utilization. Independent antimicrobial stewardship actions were defined as appropriate prescribing decisions performed without involvement by the infectious diseases pharmacists and were prospectively documented by the ASP using a Microsoft Form (Figure 1).

### Prescriber recognition

Prescribers identified as demonstrating independent appropriate stewardship practices were only recognized quarterly, and not in the moment of their actions. Recognition was conveyed by our ASP through a brief email sent to all recognized prescribers using bcc. The email summarized the service lines, intervention categories and infection types associated with antimicrobial stewardship best-practices for that quarter. A quarterly winner was selected at random, met with our ASP, and was formally acknowledged in both the quarterly email and during the following ASP committee meeting.

### Prescriber survey

To assess prescriber perceptions, an anonymous electronic survey was distributed 15 months following implementation. Eligible participants included inpatient attending physicians, residents/fellows, and advanced practice providers (APPs) recognized by the ASP. The survey assessed the initiative’s relevance, impact on stewardship awareness, acceptability of recognition, and perceived influence on prescribing behaviors. Responses were collected using a Likert-scale and free-text fields. Participation was voluntary, and no incentives were provided.

### Data collection and analysis

Descriptive data were collected on the number and types of independent stewardship actions identified and the number of prescribers recognized. Survey responses were summarized using descriptive statistics. This project was reviewed by the institutional review board and determined to be quality improvement, per institutional policy.

## Results

A total of 222 independent prescriber actions were recognized by the ASP in 2025. Results are depicted in the Table 1 below. General medicine (32%), infectious diseases (24%) and hospitalist (16%) services were most recognized. The most common intervention categories were discontinuation or avoidance of unnecessary antibiotics (53%) and treatment duration and/or IV-to-PO (22%). Asymptomatic bacteriuria (ASB) and pneumonia were the most common infection types, present in 39% and 37% of patients, respectively. ASB and suspected pneumonia later ruled out (eg, pneumonitis, congestive heart failure exacerbation) accounted for 73% (86/118) and 17% (20/118), respectively, of reasons prescribers appropriately discontinued or avoided unnecessary antibiotic use. Pneumonia was the most common infection associated with order set utilization (100% [18/18]), appropriate treatment duration or IV-to-PO transition (69% [33/48]) without order set utilization, and appropriate empiric antibiotic selection (63% [10/16]). Bloodstream infection was the most common type of infection associated with antibiotic de-escalation based on cultures (56% [9/16]). There were fewer than 10 recognized actions in each of the following: skin and soft tissue infections, urinary tract infections, empiric/sepsis, intraabdominal infections, *Clostridioides difficile* infections, bone and joint infections, or central nervous system infections.

**Table 1.** Independent prescriber actions recognized by antimicrobial stewardship pharmacists Table 1 long description.

Characteristic	Total (N = 222)
Service line, n (%)	
General medicine	72 (32)
Infectious diseases	54 (24)
Hospitalists	36 (16)
Hematology/oncology	20 (9)
Family medicine	14 (6)
Critical care medicine	11 (5)
Pulmonology	8 (4)
Surgery	7 (3)
Intervention category, n (%)	
Discontinuation/avoidance of unnecessary antibiotic	118 (53)
Treatment duration and/or IV-to-PO	48 (22)
Order set utilization	18 (8)
Antibiotic de-escalation based on culture	16 (7)
Antibiotic selection	16 (7)
Transitions of care	4 (2)
Diagnostic stewardship	1 (0)
Other	1 (0)
Infection category, n (%)	
Asymptomatic bacteriuria	86 (39)
Pneumonia	83 (37)
Bloodstream infection	17 (8)
Skin and soft tissue infection	7 (3)
Urinary tract infection	7 (3)
Intraabdominal infection	6 (3)
Empiric	6 (3)
Other	4 (2)
Not documented	4 (2)
* Clostridioides difficile* infection	1 (1)
Bone/joint	0 (0)

A total of 126 unique prescribers were identified, with 86 (68%) only being recognized once. The survey was emailed to 112 non-infectious diseases prescribers. Infectious diseases prescribers were not included to avoid biasing responses to questions pertaining to awareness of our ASP. The response rate was 29% (32/112). Most respondents were residents/fellows (47%), followed by attending physicians (28%) and APPs (25%). Prior to receiving a recognition email, most prescribers were aware the hospital had an ASP (78%).

Prescribers reported that the recognition increased their awareness of different ways to participate in antimicrobial stewardship: 25% (8/32) strongly agreed, 44% (14/32) agreed, and 31% (10/32) neutral. No prescribers disagreed or strongly disagreed. When asked if they more intentionally considered stewardship in their prescribing decisions, 19% (6/32) strongly agreed, 44% (14/32) agreed, 28% (9/32) neutral, 9% (3/32) disagreed, and no prescribers strongly disagreed.

Most prescribers felt that the antimicrobial stewardship recognition fostered a more positive relationship with the ASP (81% [26/32]), while a small number were unsure (19% [6/32]) and no one reported that it did not. Lastly, 88% (28/32) recommended continuing the initiative, 9% (3/32) were unsure, and 3% (1/32) disagreed.

## Discussion

Unlike traditional prospective audit and feedback, which targets opportunities for antimicrobial optimization, our initiative was unique in that it recognized and positively reinforced stewardship-aligned prescribing decisions, while using a low effort approach integrated into daily ASP workflow.^1^ Approximately 80% of prescribers felt that the antimicrobial stewardship recognition fostered a more positive relationship with ASPs, which may help address the perception that traditional stewardship strategies are primarily corrective or restrictive.^3,4^

This intervention also increased awareness of both our ASP existence and of different ways to participate in antimicrobial stewardship. We postulate that the quarterly results summarizing interventions broadened their understanding by providing concrete examples of how stewardship principles can be applied in routine clinical practice. Our survey results suggest that recognition-based interventions paired with traditional strategies may increase awareness, strengthen engagement, and promote a more supportive culture of antimicrobial stewardship.

Our initiative has several limitations. First, independent interventions were recognized only when encountered during routine ASP review, as described in methods. While these alerts ensured review of most patients receiving broad-spectrum antimicrobials, gaps remained, notably patients treated with narrow-spectrum agents for asymptomatic bacteriuria, those started on ampicillin/sulbactam or ceftriaxone for non-CAP indications, and patients for whom unnecessary antibiotics were either never started or rapidly discontinued. As a result, good stewardship practices that either did not trigger ASP review or for which ASPs did not log a form were not captured. Our findings thus likely underestimate the true prevalence of independent appropriate antimicrobial prescribing occurring across the hospital. Lastly, we did not assess the initiative’s impact on rates of antimicrobial utilization, or individual antibiotic prescribing patterns.

In conclusion, the antimicrobial stewardship recognition initiative, incorporating positive feedback, provided a novel complement to traditional strategies by recognizing appropriate prescribing, increasing awareness of stewardship opportunities, and fostering positive relationships between prescribers and the ASP.

## Table 1. Long description

A table with three columns and twelve rows detailing characteristics and totals of independent prescriber actions recognized by antimicrobial stewardship pharmacists. The columns are labeled Characteristic, Service line, and Intervention category. The rows list various service lines and intervention categories along with their respective counts and percentages. The table includes data on general medicine, infectious diseases, hospitalists, hematology/oncology, family medicine, critical care medicine, pulmonology, and surgery. It also details intervention categories such as discontinuation/avoidance of unnecessary antibiotics, treatment duration and/or IV-to-PO, order set utilization, antibiotic de-escalation based on culture, antibiotic selection, transitions of care, diagnostic stewardship, and other. The infection category section lists asymptomatic bacteriuria, pneumonia, bloodstream infection, skin and soft tissue infection, urinary tract infection, intraabdominal infection, empiric, other, not documented, Clostridioides difficile infection, and bone/joint.

Navigate back to Table 1..

## References

[1] Barlam TF , Cosgrove SE , Abbo LM , et al. Implementing an antimicrobial stewardship program: guidelines by the Infectious Diseases Society of America and the Society for Healthcare Epidemiology of America. Clin Infect Dis 2016;62;e51–e77. 10.1093/cid/ciw118 PMC5006285 27080992

[2] Centers for Disease Control and Prevention. The Core Elements of Hospital Antibiotic Stewardship Programs. CDC; 2019. https://www.cdc.gov/antibiotic-use/core-elements/hospital.html. 10.1093/cid/ciu542 PMC6521960 25261548

[3] Tjilos M , Drainoni ML , Burrowes SAB , et al. A qualitative evaluation of frontline clinical perspectives toward antibiotic stewardship programs. Infect Control Hosp Epidemiol. 2023;44:1995–2001. 10.1017/ice.2023.35 PMC10755145 36987859

[4] Barlam TF , Childs E , Zieminski SA , et al. Perspectives of physician and pharmacist stewards on successful antibiotic stewardship program implemention: a qualitative study. Open Forum Infect Dis 2020;7:ofaa229. 10.1093/ofid/ofaa229 PMC7367692 32704510

[5] Lin KT , Werner K , Witter S , et al. Invidual performance-based incentives for healthcare workers in Organisation for Economic Cooperation and Development member countres: a systemic literature review. Health Policy 2022;126:512–521. 10.1016/j.healthpol.2022.03.016 35422364

[6] Pouly E , Coppry M , Rogues AM , Dumartin C. Systemic review of factors promoting behaviour change toward antibiotic use in hospitals. Clin Microbiol Infect 2022;28:911–919. 10.1016/j.cmi.2022.01.005 35065264

[7] Meeker D , Linder JA , Fox CR , et al. Effect of behavioral interventions on inappropriate antibiotic prescribing among primary care practices: a randomized clinical trial. JAMA 2016;315:562–570. 10.1001/jama.2016.0275 PMC6689234 26864410

[8] Lawton R , Taylor N , Clay-Williams R , Braithwaite J. Positive deviance: a different approach to achieving patient safety. BMJ Qual Saf 2014;23;880–883. 10.1136/bmjqs-2014-003115 PMC4215344 25049424

